# Ageing and long-term smoking affects KL-6 levels in the lung, induced sputum and plasma

**DOI:** 10.1186/1471-2466-11-22

**Published:** 2011-05-11

**Authors:** Nobuhisa Ishikawa, Witold Mazur, Tuula Toljamo, Katri Vuopala, Mikko Rönty, Yasushi Horimasu, Nobuoki Kohno, Vuokko L Kinnula

**Affiliations:** 1 Department of Medicine, Pulmonary Division, University of Helsinki and Helsinki University Central Hospital, Helsinki, Finland; 2 Department of Molecular and Internal Medicine, Graduate School of Biomedical Sciences, Hiroshima University, Hiroshima, Japan; 3 Department of Medicine, Pulmonary Division, Lapland Central Hospital, Rovaniemi, Finland; 4 Department of Pathology, Lapland Central Hospital, Rovaniemi, Finland; 5 Department of Pathology, University of Helsinki and Helsinki University Central Hospital, Helsinki, Finland

## Abstract

**Background:**

KL-6 is a high-molecular-weight glycoprotein classified as a human MUC1 mucin. It was hypothesized that KL-6 could be detectable in the circulating blood and especially in airway secretions in lung diseases associated with mucus production such as chronic obstructive pulmonary disease (COPD). Additional aims of this study were to investigate whether the levels of KL-6 in plasma and sputum are related to ageing and smoking history.

**Methods:**

The concentrations of KL-6 in plasma and induced sputum supernatants from young and/or middle aged/elderly non-smokers, smokers and patients with COPD were assayed by ELISA (n = 201). The subjects were classified into five groups according to age, smoking status and presence of COPD. In addition, KL-6 expression in control and diseased lung i.e. samples from patients with COPD (n = 28), were analyzed by immunohistochemistry and digital image analysis.

**Results:**

The plasma levels of KL-6 increased with age both in non-smokers and smokers. Among middle aged/elderly subjects, plasma KL-6 levels in all smokers regardless of COPD were significantly higher than in non-smokers, whereas sputum levels of KL-6 were significantly higher in COPD compared not only to non-smokers but also to smokers. KL-6 was more prominently expressed in the bronchiolar/alveolar epithelium in COPD than in the control lungs. Plasma and sputum KL-6 levels correlated inversely with obstruction and positively with smoking history and ageing. The linear multiple regression analysis confirmed that age and cigarette smoking had independent effects on plasma KL-6.

**Conclusions:**

KL-6 increases with ageing and chronic smoking history, but prospective studies will be needed to elucidate the significance of KL-6 in chronic airway diseases.

## Background

Smokers are particularly vulnerable to suffer from chronic obstructive pulmonary disease (COPD) and several co-morbidities [1,2]. The risk of COPD increases with age and smoking history [3-5], but very little is known about the cumulative effects of ageing and long-term smoking on human lungs.

Several markers of oxidant burden and inflammation are elevated in smokers who have not developed obstruction/COPD [6-10]. However, these markers are not specific for smoking or COPD [10]. The epithelial lining fluid and mucins provide the first line of defense in the lung [11-14]. KL-6 is high-molecular-weight mucus glycoprotein also classified as a human MUC1 mucin [15-17]. Purified KL-6 can be detected by anti-MUC1 core protein antibody [16,17], and these studies have concluded that KL-6 is one subtype of the MUC1 glycoprotein. Serum KL-6 has been reported to represent a sensitive biomarker for interstitial lung diseases (ILDs) [18-20], but presumably KL-6 can be detectable also in airway secretions especially in disorders associated with mucus production such as COPD.

To test this hypothesis, the levels of KL-6 were assayed from plasma and induced sputum samples from European i.e. Finnish young and middle aged/elderly non-smokers and smokers and patients with COPD, and the distribution/expression of KL-6 was investigated by immunohistochemistry and image analysis in the control and diseased lung.

## Methods

### Subjects

Plasma and induced sputum samples from middle aged/elderly subjects were collected from subjects who had been contacted from Lapland Central Hospital [5]. Young smokers and non-smokers were military draftees from Northern Finland [21]. Details of these cohorts have been described [5,21]. Based on self-reported detailed questionnaire, all the subjects considered themselves as healthy, they had no other environmental exposures (second hand smoke, pollutants or asbestos fibers) [5]. The diagnosis of COPD was defined according to the Global Strategy for the diagnosis, management and prevention of COPD (GOLD) criteria; FEV_1 _<80% of predicted, FEV_1_/FVC <70% and bronchodilatation effect <12% [2,22,23]. Current young smokers and non-smokers had no airway obstruction (post-bronchodilator FEV_1_/FVC > 70%) and no significant reversibility. Exclusion criteria included allergies, asthma, a history of respiratory disease, or a respiratory infection less than 8 weeks before entering the study. Diagnosis of COPD in the plasma and sputum studies was confirmed during the study. None of these subjects had any previously prescribed medication for COPD or other diseases; and all the smokers were current smokers.

Tissue samples were collected from patients treated in Helsinki University Central Hospital. All control tissue samples and COPD cases were obtained from the operations of hamartomas, local lung tumors or lung transplantations. Five mg oral prednisolone and/or inhaled corticosteroids had been included in the therapy of all patients with Stage III-IV (severe-very severe) COPD, whereas none of the other subjects were receiving corticosteroid therapy. The Ethics Committees of the Helsinki University Central Hospital and Lapland Central Hospital approved the study and all patients signed written information to use the samples.

### Plasma samples

Peripheral whole venous blood was collected into EDTA tubes. Plasma was prepared by centrifugation for 10-15 min at 1,500 × *g *and stored at - 80 C until analysed.

### Induced sputum

Sputum was induced by inhalation of hypertonic saline and treated with dithioerythritol (DTE, Sigma, Germany) as recommended by the European Respiratory Society Task Force [6,24]. For the differential cell count the sample was smeared over glass slides, fixed and stained with Papanicolau stain and examined in a light microscope at 1000× magnification [25,26]. The supernatant was frozen at -80°C prior to the analyses.

### Measurement of KL-6 levels in plasma and induced sputum

KL-6 levels in plasma and induced sputum were measured by sandwich-type enzyme-linked immunosorbent assay (ELISA) using an Eitest KL-6 ELISA kit (a kind gift from Sanko Junyaku, Tokyo, Japan) according to the manufacturer's protocol as described [27]. In brief, the range of KL-6 detection is 201-4020 U/mL and the range of the standard antigen 1-20 U/mL when this kit is used by the dilution ratio 201-fold. For samples containing antigen concentrations of less than 201 U/ml, concentration can be obtained in the range of 3-600 U/ml by changing the dilution from 201 to 3-fold. Then, the detection limit of KL-6 in this study was 3 U/ml (ranging from 3-4020 U/ml).

### Immunohistochemistry and image analysis

Four mm thick paraffin-embedded tissue sections were deparaffinized, and antigens retrieved by heating the sections in citrate buffer (pH 6.0). NovoLink polymer detection system (RE7150-CE, Novocastra Laboratories ltd, Newcastle Upon Tyne, UK) was used for immunostaining according to the manufacturer's instructions. Monoclonal antibody to KL-6 (a kind gift from Sanko Junyaku, Tokyo, Japan) was used at 10 μg/ml. To determine the specificity of the staining, negative control sections were treated with mouse isotype control (Zymed Laboratories, San Francisco, CA, USA) or PBS. Two or three representative images from the lung parenchyma of each stained section were taken with an Olympus U-CMAD3 camera (Olympus Corporation, Japan) and QuickPHOTO CAMERA 2.1 software (Promicra, Praque, Czech Republic). Quantitative image analysis of the stained tissue sections was conducted as described [28]. The areas of positively vs negatively stained bronchiolar/alveolar epithelium, interstitium, and macrophages were measured with Image-Pro Plus 6.1. software (Media Cybernetics, UK).

### Statistical analysis

Data are presented as the mean ± SEM. The data were analyzed with a statistical software package (SPSS for Windows, version 15.0; SPSS Inc; Chicago, IL) and P < 0.05 was considered to indicate a significant difference. Data for individual variables from the various groups were first analyzed by the Kruskal-Wallis test followed by the Mann-Whitney U-test. Linear and multivariate regression analysis was conducted to study the independent effect of age, body mass index (BMI), smoking status and COPD on KL-6.

## Results

### Plasma levels of KL-6 increase with ageing being highest in chronic smokers and patients with COPD

**Figure 1:**
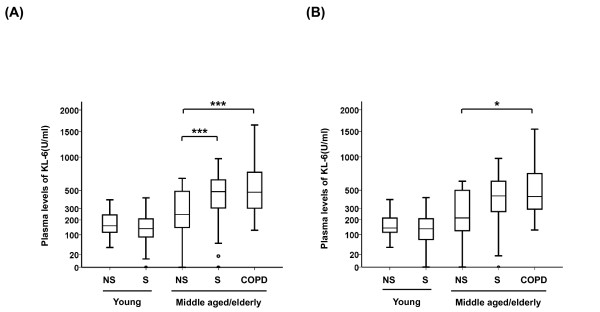
**KL-6 levels in plasma**. KL-6 levels in plasma obtained from young subjects (non-smokers; NS and smokers; S) and middle aged/elderly subjects (non-smokers, smokers and COPD) in (A) both males and females, and in (B) males only. The box represents the 25^th ^to 75^th ^percentiles, the solid lines within the boxes show the median values, the whiskers are the 10^th ^and 90^th ^percentiles, and the points represent outliers. Horizontal bars indicate mean values. ^†^p < 0.05; ^††^p < 0.01; ^†††^p < 0.001 (young non-smokers vs young smokers, Mann-Whitney U test). *p < 0.05; **p < 0.01; ***p < 0.001 (middle aged/elderly non-smokers vs middle aged/elderly smokers or COPD, Mann-Whitney U test).

The clinical characteristics and lung function data (63 young subjects and 138 middle aged/elderly subjects) are shown in Table 1. The age of the young subjects was in the range 18-22 years and the middle aged/elderly subjects 35-79 years. Plasma levels of KL-6 in the five groups are shown in Figure 1A. The plasma levels of KL-6 increased with age both in non-smokers and smokers (p = 0.008 and p < 0.001, respectively). In the young subjects, the mean plasma levels of KL-6 did not differ between the non-smokers and smokers (mean ± SEM, young non-smoker 172 ± 12 U/ml, young smoker 160 ± 19 U/ml). In the middle aged/elderly subjects, the mean plasma level of KL-6 was higher in both smokers and subjects with COPD when compared to non-smokers (non-smoker 309 ± 35 U/ml, smoker 478 ± 31 U/ml, COPD 589 ± 61 U/ml; p = 0.003 and p < 0.001, respectively). There were more women in the middle aged/elderly group than in the young group. Therefore, these 201 middle aged/elderly subjects were divided into two subgroups by gender. Mean plasma level of KL-6 was higher in the middle aged/elderly males with COPD when compared to non-smokers in the group (middle aged/elderly male non-smokers 292 ± 78 U/ml, middle aged/elderly males with COPD 556 ± 64 U/ml; p = 0.037) (Figure 1B).

**Table 1:** Characteristics of the subjects in the plasma analyses

Variable	Young		Middle aged/elderly		
	**Non-smokers**	**Smokers**	**Non-smokers**	**Smokers**	**COPD**

Subjects, n	28	35	34	64	40
Age, yr	20 ± 0.2	20 ± 0.1	56 ± 1.7	52 ± 1.0	61 ± 1.4**
Sex, M/F	26/2	34/1	9/25	40/24**	31/9***
Pack years, yr	0	5.0 ± 0.43^†††^	0	30.5 ± 1.78***	39.6 ± 2.39***
Post bronchodilator					
FVC (l)	5.5 ± 0.17	5.4 ± 0.14	3.6 ± 0.11	4.0 ± 0.11	3.8 ± 0.15
FEV_1 _(l)	4.8 ± 0.14	4.7 ± 0.11	3.0 ± 0.09	3.3 ± 0.09	2.3 ± 0.12***
FEV_1 _(% predicted)	98 ± 1.2	100 ± 1.6	106 ± 2.3	95 ± 1.5***	71 ± 2.7***
FEV_1_/FVC	88 ± 1.0	88 ± 0.8	84 ± 1.0	83 ± 0.6	61 ± 1.5***

Data are shown as mean ± SEM

COPD, chronic obstructive pulmonary disease; FVC, Forced vital capacity; FEV_1_, Forced expiratory volume in one second

^†^p < 0.05; ^††^p < 0.01; ^†††^p < 0.001 (vs young non-smokers, Mann-Whitney U test or Chi-square test)

*p < 0.05; **p < 0.01; ***p < 0.001 (vs middle aged/elderly non-smokers, Mann-Whitney U test or Chi-square test)

### Sputum levels of KL-6 increase in COPD

**Figure 2:**
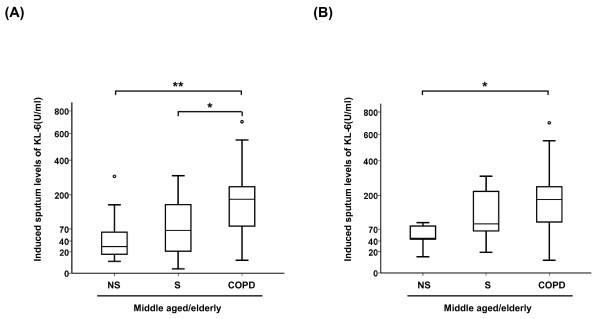
**KL-6 levels in induced sputum**. KL-6 levels in induced sputum obtained from middle aged/elderly subjects (non-smokers; NS, smokers; S and COPD) in (A) both males and females, and in (B) males only. The box represents the 25^th ^to 75^th ^percentiles, the solid lines within the boxes show the median values, the whiskers are the 10^th ^and 90^th ^percentiles, and the points represent outliers. Horizontal bars indicate mean values. *p < 0.05; **p < 0.01; ***p < 0.001 (middle aged/elderly non-smokers vs middle aged/elderly smokers or COPD, Mann-Whitney U test).

The levels of KL-6 were next investigated in the induced sputum obtained from 15 middle aged/elderly non-smokers, 20 smokers and 19 patients with COPD (Table 2). In the middle aged/elderly subjects, female gender and younger age were more predominant in non-smokers than in the COPD patients. The difference in the induced sputum levels of KL-6 between the non-smoker, smoker and COPD groups was significant (p = 0.008) (Figure 2A). The mean induced sputum level of KL-6 was higher in patients with COPD than in the middle aged/elderly smokers without COPD; 203 ± 41 U/ml vs 96 ± 22 U/ml, p = 0.002), the corresponding value in the middle aged/elderly non-smokers being 68 ± 20 U/ml, p = 0.033). Mean induced sputum level of KL-6 was higher in the middle aged/elderly males with COPD when compared to the non-smokers in the group (middle aged/elderly male non-smokers 54 ± 13 U/ml, middle aged/elderly males with COPD 232 ± 64 U/ml; p = 0.021) (Figure 2B).

**Table 2:** Characteristics of the subjects in the induced sputum analyses

Variable	Middle aged/elderly		
	**Non-smokers**	**Smokers**	**COPD**

Subjects, n	15	20	19
Age, yr	52 ± 1.9	52 ± 1.5	61 ± 1.9***
Sex, M/F	5/10	13/7	17/2**
Pack years, yr	0	29 ± 1.9***	45 ± 3.8***
Post-bronchodilator			
FVC, l	3.9 ± 0.21	4.2 ± 0.19	3.6 ± 0.15
FEV_1_, l	3.3 ± 0.15	3.5 ± 0.13	2.1 ± 0.13***
FEV_1_, % predicted	107 ± 3.9	100 ± 1.7	64 ± 3.2***
FEV_1_/FVC	86 ± 1.4	83 ± 1.0	59 ± 2.7***

Data are shown as mean ± SEM

COPD, chronic obstructive pulmonary disease; FVC, Forced vital capacity; FEV_1_, Forced expiratory volume in one second

*p < 0.05; **p < 0.01; ***p < 0.001 (vs middle aged/elderly non-smokers, Mann-Whitney U test or Chi-square test)

### KL-6 is prominently expressed in the bronchiolar/alveolar epithelium in COPD lungs

**Figure 3:**
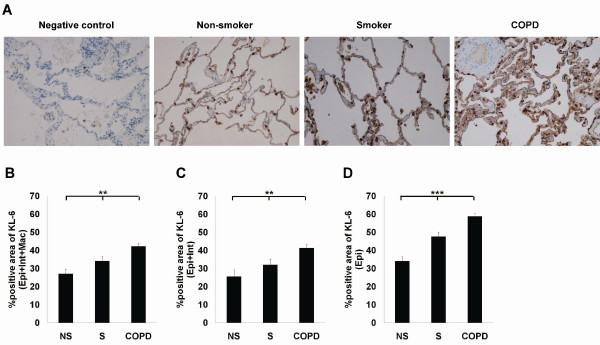
**KL-6 expression and localization in diseased lung**. (A) KL-6 expression and localization in representative sections of lung specimens from non-smoker, smoker, and patient with COPD. Positive KL-6 expression was seen mainly in type II pneumocytes as well as in macrophages in the lungs of non-smokers, smokers, and patients with COPD. The bronchial/alveolar epithelium of patients with COPD displayed highly positive areas of KL-6 staining in contrast to the situation in non-smokers and smokers. (B) Quantitative image analysis of KL-6 in the lung tissues of 7 non-smokers, 7 smokers and 14 patients with COPD. Three representative areas consisting of the parenchymal portion of the lung tissue were analyzed from all stained sections (sum of the bronchiolar/alveolar epithelium, interstitium and macrophages; Epi+Int+Mac). Quantitative image analysis of the immunoreactivity for KL-6 was also conducted separately in the bronchial/alveolar epithelium and interstitium (sum of the bronchiolar/alveolar epithelium and interstitium; Epi+Int; C) or the bronchial/alveolar epithelium (sum of the bronchiolar/alveolar epithelium; Epi; D). Data are presented as mean ± SEM. *p < 0.05; **p < 0.01; ***p < 0.001 (between all four groups, Kruskall-Wallis test). For patient characteristics see Table 3.

Next, the distribution of KL-6 in the lung tissue was investigated in 7 non-smoking controls, 7 smokers and 14 patients with COPD (Table 3). In agreement with earlier published data [15,18], KL-6 could be detected in the alveolar type II cells and macrophages in all lung specimens (Figure 3A). In addition, KL-6 was expressed in the bronchiolar/alveolar epithelial cells and interstitium in COPD. Since KL-6 has not been evaluated in COPD lungs earlier, the distribution and quantitation was conducted by using digital image analysis. There was significant difference in the KL-6 positive areas (sum of the bronchiolar/alveolar epithelium, interstitium, and macrophages; Epi+Int+Mac, respectively) between the three groups (p = 0.005) (Figure 3B). Patients with COPD displayed higher KL-6 positive area in general (Epi+Int+Mac) compared to the findings in the lungs in non-smokers or smokers (p < 0.001 and p = 0.010, respectively). KL-6 was also expressed in macrophages, which are phagocytic cells. When macrophages were excluded from these calculations (sum of the bronchiolar/alveolar epithelium and interstitium; Epi+Int), the difference among the three groups remained significant (p = 0.002) (Figure 3C). When the KL-6 positive area in bronchiolar/alveolar epithelium was evaluated but excluding macrophages and interstitium (Epi), the difference among the three groups was still significant (p < 0.001). Smokers, as well as the patients with COPD displayed higher KL-6 positive bronchiolar/alveolar epithelium (sum of the bronchiolar/alveolar epithelium; Epi) than non-smoker's lung (p = 0.006 and p < 0.001, respectively), and the epithelial positivity was more prominent in the COPD than in the smoker's lung (Figure 3D).

**Table 3:** Characteristics of the controls and subjects with COPD in the immunohistochemical analyses of the lung

	Non-smokers	Smokers	COPD
Subjects, n	7	7	14
Age, yr	65 ± 3.1	62 ± 2.6	60 ± 2.1
Sex, M/F	3/4	6/1	9/4
Pack years, yr	- ^†^	24 ± 5.7	37 ± 3.8
Post-bronchodilator			
FVC, l	4.5 ± 0.56	3.7 ± 0.25	2.4 ± 0.31**
FEV_1_, l	3.5 ± 0.47	3.1 ± 0.19	1.2 ± 0.24**
FEV_1_, % predicted	106 ± 4.6	88 ± 3.5*	34 ± 6.1***
FEV_1_/FVC	79 ± 2.8	82 ± 2.3	45 ± 5.3**

Data are shown as mean ± SEM

COPD, chronic obstructive pulmonary disease; FVC, Forced vital capacity; FEV_1_, Forced expiratory volume in one second

^† ^Two of the controls were never smokers and four of the controls had smoked for between 10-30 years but had stopped smoking at least 2 years before the study.

*p < 0.05; **p < 0.01; ***p < 0.001 (vs non-smokers, Mann-Whitney U test or Chi-square test)

### KL-6 levels correlate inversely with obstruction and positively with smoking history

The correlations between the KL-6 levels and several clinical parameters including age, smoking status and lung function values are shown in Tables 4 and 5. A linear regression analysis confirmed that age (regression coefficient (B) = 8.178, standard error (SE) = 0.960, p < 0.001), BMI (B = 12.879, SE = 5.285, p = 0.016), Pack-year (B = 6.765, SE = 0.939, p < 0.001) and FEV_1_/FVC (B = -8.507, SE = 1.684, p < 0.001) had significant effects on the KL-6 levels in plasma (Table 4), and that age (B = 4.747, SE = 2.360, p = 0.049), Pack-year (B = 2.921, SE = 0.942, p = 0.003) and FEV_1_/FVC (B = -0.3034, SE = 1.415, p = 0.037) had significant effects on the KL-6 levels in induced sputum (Table 5). In addition, multivariate regression analyses demonstrated that age (B = 6.383, SE = 1.262, p < 0.001) and pack-year (B = 3.832, SE = 1.251, p = 0.002) had independent significant effects on plasma KL-6 (Table 4).

**Table 4:** Linear and multivariate analysis of the relationship between the plasma levels of KL-6 and clinical parameters

Variable		Regression coefficient	Standard error	P-value
Linear Regression	Age	8.178	0.960	<0.001***
	BMI	12.879	5.285	0.016*
	Pack-year	6.765	0.939	<0.001***
	FEV_1_/FVC	-8.507	1.684	<0.001**

Multi Regression	Age	6.383	1.262	<0.001***
	BMI	-3.464	4.999	0.489
	Pack-year	3.832	1.251	0.002**
	FEV_1_/FVC	1.008	2.054	0.624

BMI, body mass index; FEV_1_, Forced expiratory volume in one second; FVC, Forced vital capacity

**Table 5:** Linear and multivariate analysis of relationship between the induced sputum levels of KL-6 and clinical parameters

Variable		Regression coefficient	Standard error	P-value
Linear Regression	Age	4.747	2.360	0.049*
	BMI	5.971	5.427	0.276
	Pack-year	2.921	0.942	0.003**
	FEV_1_/FVC	-3.034	1.415	0.037*

Multi Regression	Age	1.506	2.713	0.581
	Pack-year	2.566	1.409	0.075
	FEV_1_/FVC	-0.056	1.940	0.977

BMI, body mass index; FEV_1_, Forced expiratory volume in one second; FVC, Forced vital capacity

## Discussion

The present study supports the hypothesis that KL-6 is associated with the pathogenesis of cigarette smoke induced lung damage and COPD. Plasma levels of KL-6 were found to be elevated with age both in non-smokers and smokers. In addition, in middle aged/elderly subjects, plasma levels of KL-6 in all smokers were higher than in non-smokers. A multivariate regression analysis demonstrated that KL-6 was clearly correlated with age and pack-years. One important new finding was the higher level of KL-6 in the sputum samples of COPD patients compared not only to non-smokers but also to those found in smokers. KL-6 was more prominently expressed in the bronchiolar/alveolar epithelium in COPD lungs than in non-smokers' and smokers' lung. As far as we are aware, this is the first study on KL-6 distribution/expression in human lung tissues and the effects of ageing and long-term smoking on KL-6 levels in plasma and induced sputum obtained from a non-Asian population over a wide age range.

KL-6 is a high-molecular weight (>200 kd) mucus glycoprotein, which belongs to MUC1 protein [15-17]. Chronic productive cough is associated with mucus hypersecretion and airway inflammation [29]. Mucins provide significant protection against oxidants, and the levels of these glycoproteins are increased by exposure to oxidants [30]. KL-6/MUC1 expression is also elevated by oxidative stress, and MUC1 in turn induces the expression of several anti-oxidant enzymes, and attenuates apoptotic response to oxidative stress [31]. It is therefore surprising that KL-6 has not been earlier evaluated from the circulating blood or from airway secretions of smokers and/or patients with COPD.

Another new finding from this study was that among middle aged/elderly subjects, when compared to non-smokers, the plasma KL-6 level was already elevated in smokers regardless of COPD diagnosis. This difference could not be seen in young smokers. This finding is in agreement with earlier studies that have reported serum levels of KL-6 correlating with age but not smoking history among healthy young/middle aged Caucasian subjects [27,32]. An additional strength of this study is that the subjects included in the plasma and sputum studies had no history of exposures to other agents and no co-morbidities and medications.

The circulating KL-6 levels were higher than those earlier reported in Japanese control subjects [18,33]. KL-6 levels were studied from plasma, whereas Japanese values have been published from serum. On the other hand, an earlier study has demonstrated that serum and plasma levels of KL-6 are very similar [34]. A recent study on the functional A to G MUC1 gene polymorphism at nucleotide position 568 (exon 2; rs4072037) and the variation of serum levels of KL-6 in Caucasian subjects, showed that the highest serum KL-6 levels can be detected in the GG genotype, the lowest levels in AA genotype with intermediate levels in the AG genotype [32]. According to the HapMap data http://hapmap.ncbi.nlm.nih.gov/, the genotype frequency for AA, AG and GG genotype in European subjects (30.1%, 55.8% and 14.2%, respectively) differs from Japanese subjects (69.8%, 25.6% and 4.7%, respectively). Overall, the cut-off level for KL-6 in the European is probably higher than in the Asian/Japanese population but will require further investigations.

Mucins have an important role in the pathogenesis of chronic airway disease [35]. In this study, the levels of KL-6 in induced sputum were comparable with those in circulating blood, indicating that an extremely large volume of KL-6 exists in the ELF of the patients with COPD. Furthermore, sputum levels of KL-6 were higher in COPD compared not only to middle aged/elderly non-smokers but also to smokers. Based on these new findings, the level of KL-6 in induced sputum may be more sensitive and more specific than KL-6 in the circulating blood for the evaluation of COPD.

Sputum levels of KL-6 were elevated in patients with COPD but not in "healthy" smokers or non-smokers. Induced sputum reflects mainly large airway pathology. KL-6 was mainly localized in bronchial/alveolar epithelium and alveolar macrophages both in the healthy and diseased lung, and significantly elevated in specific cell types in smokers and/or COPD even though the number of the cases was relatively low. Since KL-6 is a high molecular weight glycoprotein, both alveolar-capillary destruction and enhancement of alveolar-capillary permeability are probably needed for the elevation of circulating KL-6 related molecules [36]. Taken together, it can be concluded that sputum KL-6 could be good biomarker for the injury in distal airways, but it does not reflect smoking induced alterations in the airways of "healthy" lung.

There were some limitations. First, the sample size was relatively small. Secondly, there was individual variability between the subjects, especially in the sputum levels of KL-6, which is in line with previous studies showing that the repeatability of sputum in longitudinal studies is, at best, only satisfactory [37]. Thirdly, we did not perform high resolution computed tomography (HRCT) scan, which could give more information about the COPD sub-phenotypes. Patients with combined pulmonary fibrosis and emphysema (CPFE) are not very uncommon, and these particular patients have high levels of circulating KL-6 [38]. In spite of these concerns, the results are straightforward and significant. Considering ethnic differences in the prevalence of α-1-antitrypsin (AAT) deficiency and the MUC polymorphism (rs4072037) [39], the results need to be cautiously interpreted for Asian/Japanese patients. This was also a cross-sectional study, and therefore the validation and definition of the specificity of these proteins in various chronic airway diseases will also require further investigations. Prospective follow-up is currently ongoing in two Finnish cohorts, which contain smokers and patients with COPD [5,40].

## Conclusions

In conclusion, ageing alone and/or long-term smoking appears to lead to an increase in plasma and sputum KL-6, but prospective studies will be needed to elucidate the significance of these findings in the development and progression of COPD.

## Abbreviations

AAT: α-1-antitrypsin; B: regression coefficient; COPD: chronic obstructive pulmonary disease; ELISA: enzyme-linked immunosorbent assay; FVC: forced vital capacity; FEV1: forced expiratory volume in one second; HRCT: high resolution computed tomography; KL-6: Krebes von den lungen-6; SD: standard deviation; SEM: standard error of the mean;

## Competing interests

Nobuoki Kohno has a personal royalty of KL-6 from a Japanese pharmaceutical company, Eisai Co., Ltd. The remaining authors have no conflicts of interest.

## Authors' contributions

NI participated in the design of the study, analyzed the laboratory analysis, performed part of the statistical analysis and drafted the manuscript. WM prepared the figures and participated in the writing process. TT participated in the recruitment and interview of the subjects and their characterization and was responsible for the lung function analyses. KV and MR participated in the evaluation of induced sputum and tissue immunohistochemistry. YH contributed to the statistical analyses and interpretation of data. NK participated in the design of the study. VLK conceived the study, and participated in its design and coordination, and helped to draft the manuscript. All authors have read and approved the final manuscript.

## Authors' information

^1 ^Department of Medicine, Pulmonary Division, PO Box 22 (Haartmaninkatu 4), FI-00014 University of Helsinki and Helsinki University Central Hospital, Helsinki, Finland

^2 ^Department of Molecular and Internal Medicine, Graduate School of Biomedical Sciences, Hiroshima University, 1-2-3 Kasumi, Minami-ku, Hiroshima 734-8551, Japan

^3 ^Department of Medicine, Pulmonary Division, Lapland Central Hospital, Ounasrinteentie 22, FI-96101, Rovaniemi, Finland

^4 ^Department of Pathology, Lapland Central Hospital, Ounasrinteentie 22, FI-96101, Rovaniemi, Finland

^5 ^Departments of Virology and Pathology, Haartman Institute, PO Box 21 (Haartmaninkatu 3), FI-00014 University of Helsinki, Helsinki, Finland

## Pre-publication history

The pre-publication history for this paper can be accessed here:

http://www.biomedcentral.com/1471-2466/11/22/prepub
